# Current Status of the Third-Line *Helicobacter pylori* Eradication

**DOI:** 10.1155/2018/6523653

**Published:** 2018-05-02

**Authors:** Jae Ho Choi, Young Joo Yang, Chang Seok Bang, Jae Jun Lee, Gwang Ho Baik

**Affiliations:** ^1^Institute of New Frontier Research, Hallym University College of Medicine, Chuncheon, Republic of Korea; ^2^Department of Internal Medicine, Hallym University College of Medicine, Chuncheon, Republic of Korea; ^3^Department of Anesthesiology and Pain Medicine, Hallym University College of Medicine, Chuncheon, Republic of Korea

## Abstract

Antibiotic resistance is growing worldwide, and patients who have failed consecutive 1st- and 2nd-line *H. pylori* eradication regimens are increasing. Therefore, the role of the bacterial culture with antibiotic susceptibility testing and molecular susceptibility testing is important for avoiding the use of ineffective antibiotics. However, antibiotic susceptibility testing-guided treatment does not necessarily guarantee successful eradication, and there have been mixed results for the effectiveness of a 3rd-line rescue therapy. Therefore, providing patients with pretreatment medication instructions and education is important. It is also crucial to determine the reason of the eradication failure, including host-related factors (poor compliance to eradication regimen, smoking, and cytochrome P450 2C19 genetic polymorphism) or treatment-related factors (inadequate dosage or duration of therapy and gastric acidity), as such factors can be modified for a tailored therapy. Although the indications for *H. pylori* eradication have widened, patients at a high risk of gastric cancer can gain definitive benefits with a 3rd-line or even 4th-line therapy.

## 1. Introduction

*Helicobacter pylori* (*H. pylori*) is associated with various gastrointestinal diseases or conditions, such as gastritis, peptic ulcers, mucosa-associated lymphoid tissue (MALT) lymphoma, gastric adenocarcinoma, and even dyspepsia [1, 2]. Extraintestinal associations of *H. pylori* have also been investigated, including immune thrombocytopenic purpura, unexplained iron deficiency anemia, or vitamin B12 deficiency [3–5]. The elimination of this pathogen can cure the associated diseases or alter their clinical course, although the impact is dependent on each resultant disease or condition [2, 6]. Therefore, expert consensus guidelines suggest that *H. pylori* should be considered and treated as an infection irrespective of clinical symptoms [2, 7]. Although only 1–15% of patients with *H. pylori* infection develop clinical disease, even asymptomatic *H. pylori*-associated gastritis should be treated to prevent severe complications, such as gastric cancer, as reported by the Kyoto Global Consensus Conference suggesting a paradigm shift from treatment to prevention [6, 7]. This strategy is often misleading but is recommended in regions with high incidence of gastric cancer.

There are varying perspectives in the management of *H. pylori* infection as a preventive strategy. Previous studies in areas with high prevalence of gastric cancer have focused on the role of *H. pylori* eradication on gastric cancer prevention [8]. Such benefits have been consistently emphasized, but the evidence regarding the harmful effects of *H. pylori* eradication has been scarce and hardly emphasized. An example is the concept of symbiosis (mutualism) between *H. pylori* and humans and the inverse correlation between *H. pylori* infection and the prevalence of esophageal cancer [9]. Mass eradication is likely to cause dysbiosis of the human gut microbiome and has the possibility to cause resultant diseases or conditions [10]. Rising antimicrobial resistance is another concern because specific point mutations in the DNA of *H. pylori* caused by antibiotic misuse are the main molecular mechanism underlying drug resistance [11].

Since antibiotic resistance is rapidly growing worldwide, patients who have failed consecutive 1st- and 2nd-line eradication regimens are increasing. Currently, the best treatment strategy for these patients is prescribing susceptible antibiotics based on the results of a bacterial culture with susceptibility testing (detecting phenotype resistance using agar dilution method as a gold standard). Recently, molecular susceptibility testing has become an alternative method, which allows rapid identification of resistance-associated mutations (e.g., point mutations in 23S rRNA for clarithromycin resistance or *gyrA* for levofloxacin resistance) [12, 13]. These methods can prevent unpredictable side effects and development of antibiotic resistance by avoiding unnecessary use of antibiotic combinations [14].

However, these methods for testing are not routinely and widely available. Moreover, there are also resistance-associated point mutations whose clinical meaning is unknown yet. Therefore, an empiric antibiotic combination is widely recommended for the eradication of *H. pylori* [15]. Without knowing the antibiotic resistance profile for *H. pylori*, there is a possibility of failure of 1st- and 2nd-line eradiation regimens. In terms of 3rd-line eradication, there are a few data on how many physicians are willing to prescribe and whether it is mandatory or optional. In this review, the authors will discuss the pros and cons of 3rd-line *H. pylori* eradication.

## 2. Currently Recommended 3rd-Line Eradication Methods

Although many empiric rescue therapies have been attempted following the failure of the 1st- and 2nd-line *H. pylori* eradication regimens, according to the Maastricht V/Florence Consensus Report, a bacterial culture with susceptibility testing or molecular testing for genotype resistance-guided treatment is recommended whenever possible [2]. Although susceptibility testing-guided treatment is theoretically superior to empiric treatment, comparative efficacy has been proven only in the 1st-line or 2nd-line treatment [2, 16–18]. It is impossible to perform unethical clinical trials comparing the efficacy of these therapies because, theoretically, susceptibility testing-guided treatment will always be superior to empiric treatment [19].

The routine laboratory examination of bacterial culture with susceptibility testing is not available in all institutions [15]. Therefore, an empiric regimen reflecting the most likely clinical situation is also recommended, which, according to the Maastricht V/Florence Consensus Report (in regions of low clarithromycin resistance), is a fluoroquinolone-containing regimen after failure of a clarithromycin-containing 1st-line and bismuth-containing quadruple 2nd-line regimens [2]. Due to the rising resistance to fluoroquinolones, a rifabutin-containing therapy or a combination of bismuth with different antibiotics is recommended in regions with high fluoroquinolone resistance [2].

After failure of the 1st-line treatment with non-bismuth-containing quadruple therapy (in regions of high clarithromycin resistance and low-to-intermediate metronidazole resistance) and 2nd-line treatment with a fluoroquinolone-containing regimen, the bismuth-containing quadruple therapy can be recommended as a 3rd-line therapy [2, 20, 21].

In cases where the bismuth-containing quadruple therapy (in regions of high clarithromycin and metronidazole resistance) was used as a 1st-line regimen and a fluoroquinolone-containing regimen was used as a 2nd-line regimen, the abovementioned strategy remains unchanged (rifabutin-containing regimen or combination of bismuth with different antibiotics is the remaining option), and susceptibility testing-guided treatment is beginning to receive more attention [2, 15].

## 3. Unsolved Issues

The 1st issue is that a bacterial culture with antimicrobial susceptibility testing is not perfect. The culture success rate of *H. pylori* is relatively low (approximately 55–73% or less) [12]. It is also time-consuming and takes more than 72 to 96 hours for inoculation and subculture of *H. pylori* in the agar media plate [13, 22]. The cultured *H. pylori* from several samples of biopsied specimen may not accurately represent the bacteria of the entire stomach [22]. Due to differences in external factors, including incubation condition, growth media, and examination technique, the results of antimicrobial susceptibility testing may not be consistent [22, 23]. Moreover, a single in vitro antibiotic resistance profile at one time point might not reflect the efficacy of antibiotic combinations and the in vivo resistance profile [24]. The interpretation of the results is also confusing. Clarithromycin is the only drug recommended by the Clinical and Laboratory Standards Institute for drug susceptibility testing of *H. pylori*, and a uniform quality standard is not yet established [15, 25].

Susceptibility testing-guided treatment does not always guarantee successful eradication, and there have been mixed results for the effectiveness of this treatment [15, 26]. Third-line susceptibility testing-guided treatment often fails to eradicate *H. pylori* infection indicating that this test does not reflect in vivo susceptibility perfectly, and various factors are involved other than in vitro antibiotic resistance [27]. The possible factors include microorganism-related factors (high bacterial load and biofilm of *H. pylori*), host-related factors (poor compliance to eradication regimen, smoking, cytochrome P450 2C19 genetic polymorphism, and impaired mucosal immunity), or treatment-related factors (inadequate dosage or duration of therapy and gastric acidity) [22, 27, 28]. Poor adherence was reemphasized as an important cause of eradication failure after 1st-line and 2nd-line treatments [29], and extending the duration of quinolone-containing rescue therapy showed increasing eradication rate in a recent study [30]. Infection with multiple strains other than *H. pylori* is another possible reason [31, 32]; more than 2 strains were cultured in 65% of patients, and the antibiotic susceptibility profile among the different strains was consistent only in 61.1% of patients in a Korean study [32]. Therefore, molecular susceptibility testing may better represent the in vitro antibiotic resistance profile, although comparative efficacy has not been proven [27, 33]. Moreover, the cost-effectiveness of a culture with susceptibility testing to determine the 3rd-line *H. pylori* eradication regimen has not been determined [2, 15].

Another issue is that a fluoroquinolone-containing regimen is not sufficient as the 3rd-line eradication therapy. Although empiric therapy is recommended after failure of a clarithromycin-containing 1st-line and a bismuth-containing quadruple 2nd-line regimens according to the Maastricht V/Florence Consensus Report [2], antibiotic resistance is continuously rising. In Korea, the resistance rate of ciprofloxacin, levofloxacin, and moxifloxacin is reported to be up to 38.2%, 37.7%, and 34.6%, respectively, which are higher than those previously reported [34, 35]. There are no officially recommended guidelines for empiric therapy in Korea [36–39]. Considering that resistance to quinolones is acquired easily, this regimen should not be widely recommended.

Another recommended therapy is a rifabutin-containing regimen in regions with high fluoroquinolone resistance [2]. The 3rd-line rescue treatment with rifabutin-containing high-dose proton-pump inhibitor- (PPI-) combined therapy has been successful in the Korean population (eradication success of 96.3% in intention-to-treat (ITT) and 100% in per-protocol (PP) analysis) [40]. However, cross-resistance between rifabutin and rifampin is a serious concern in tuberculosis prevalent countries such as Korea due to theoretical concerns that overuse may increase the prevalence of rifabutin-resistant mycobacteria in the community [21, 41, 42].

Difficulty conducting clinical trials for a 3rd-line empirical regimen is another concern. Many patients discontinue the 3rd-line therapy because of drug-associated adverse events or concerns about antibiotic overuse. Most of the studies exploring rescue therapy have included only a small number of patients.

## 4. Are There Any Specific Indications for 3rd-Line *H. pylori* Eradication?

Since the paradigm shift of *H. pylori* infection from treatment to prevention, the indications for eradication have widened [6, 7]. In addition to the traditional indications including patients with peptic ulcers, gastric MALT lymphoma, and early gastric cancer after endoscopic resection, the following conditions were included in the Korean National Health Insurance Scheme in January 2018 to allow for individualized determination of the risk factors of gastric cancer: patients with idiopathic thrombocytopenic purpura, family history of gastric cancer, gastric adenoma after endoscopic resection, or atrophic gastritis, and patients who agree with the eradication.

As the purpose of eradication is to prevent serious complications triggered by *H. pylori* infection, the following famous passage should be considered: “the attitude in *H. pylori* eradication failure, even after two or more unsuccessful attempts, should be to fight and not to surrender” [43]. Supporting evidence for this strategy is that gastric cancer develops in *H. pylori*-infected persons but not in uninfected persons [44]. However, it is expected that patients who are not cured after 2 consecutive treatments will show at least a single or double acquired resistance to the previously used antibiotics [34, 43]. The isolates from the previous eradication failure are classified as secondary strains, and the antimicrobial resistance of these strains (2nd resistance) is higher than the primary resistance from selection of mutations or cross-resistance and subsequent horizontal transmission of mutant resistance genes related to the antibiotics used [22, 34, 45, 46]. The increase in antibiotic resistance after failure of eradication is obvious and is a significant limitation for subsequent successful eradication, which is reflected by lower success rates of the 3rd-line eradication regimens, especially for clarithromycin, metronidazole, and levofloxacin [34, 47, 48].

The cumulative eradication rate is known to reach almost 100% after 2 or more consecutive eradication failures [49]. Therefore, successive retreatments are emphasized based on the benefits of treatment [49]. However, the impact of eradication is different among patients with peptic ulcers, MALT lymphoma, early gastric cancer, and *H. pylori-*associated gastritis. Successive trials of eradication should be performed in patients at a high risk for gastric cancer. However, the risks and benefits to patients with moderate or low risk of gastric cancer should be balanced because potent antibiotics for the 3rd-line eradication are not yet available, and 3rd-line susceptibility testing-guided eradication has shown mixed results [15, 26].

## 5. What Is the Current Appropriate Method to Determine 3rd-Line Treatment, Susceptibility Testing-Guided Therapy, or an Empiric Antibiotic Combination That Can Overcome Multidrug Resistance?

The principle of salvage treatment is using two or more antibiotics that were not previously prescribed [50, 51]. Because of selection of mutations or cross-resistance after 2 consecutive eradication trials, appropriate candidates are limited. Although the susceptibility testing-guided therapy is preferred whenever possible, the empiric regimens are still warranted, and potential candidates include sitafloxacin or rifabutin. A recent study in Japan showed eradication success in 83% of cases by ITT analysis using sitafloxacin-containing 3rd-line therapy [52]. Sitafloxacin has shown better efficacy than levofloxacin and is known to be effective regardless of the *gyrA* mutation status of the *H. pylori* strains [53–55]. However, this test is not available worldwide, and resistance to quinolones is acquired easily [20, 56]. Furthermore, some strains develop strong resistance via acquisition of double mutations in *gyrA* [20, 56].

Rifabutin, a derivative of rifamycin, has a very low rate of resistance and is chemically stable over a wide pH range, and its antibacterial activity is not affected by the acidic environment of the stomach [40, 42, 57, 58]. A recent Japanese trial showed an 83.3% (10 days) to 94.1% (14 days) success rate of a rifabutin-containing 3rd- or 4th-line regimen using ITT analysis [59]. However, rifabutin is associated with serious adverse events, such as myelotoxicity (leukopenia or thrombocytopenia), and the cost is high. Although low phenotypic resistance has been reported, a valid genotypic marker other than mutations in the *rpoB* gene for rifabutin resistance is not yet available [60]. All patients with *rpoB* mutation-positive strains showed successful eradication in a Japanese study, and mycobacterial strains bearing this mutation are often rifabutin-susceptible [59, 61].

Rifaximin is a semisynthetic derivative of rifamycin for the treatment of intestinal bacterial infections. Because of the pyridoimidazole ring of this drug, it is virtually nonabsorbable and can maintain high concentrations in the gastrointestinal tract [62]. Therefore, the concentration in the blood stream is low, and the drug does not have the serious adverse events associated with rifabutin. However, therapeutic concentrations within the gastric mucus layer are low, and the eradication rate as a 3rd-line regimen (rifaximin 200 mg t.i.d., levofloxacin 500 mg q.d., and lansoprazole 15 mg b.i.d. for 1 week) showed suboptimal results (65% eradication success after failure of the clarithromycin-containing triple therapy and bismuth-containing quadruple therapy) [63].

After failure of the 1st-line treatment with the non-bismuth-containing quadruple therapy (in regions of high clarithromycin resistance and low-to-intermediate metronidazole resistance) and 2nd-line treatment with a fluoroquinolone-containing regimen, the bismuth-containing quadruple therapy can be recommended as a 3rd-line therapy [2, 20, 21]. However, the bismuth-containing quadruple therapy is recommended as a 1st-line regimen in regions of high dual clarithromycin and metronidazole resistance [2]. Modified quadruple therapy (14-day therapy with b.i.d. lansoprazole 30 mg and bismuth 220 mg, plus metronidazole 400 mg q.i.d and amoxicillin 1 g t.i.d) was compared with the bismuth-containing quadruple therapy in patients who had previously failed two or more courses of eradication therapies that included amoxicillin, clarithromycin, nitroimidazole, or a fluoroquinolone in China [64]. This randomized controlled trial showed noninferior efficacy of modified quadruple therapy (ITT: 88.5%, PP: 93.7%) (versus bismuth-containing quadruple therapy), and this could be a candidate regimen for rescue therapy [64].

Based on the low resistance rate of amoxicillin, high-dose PPI and amoxicillin dual therapy are often recommended as an alternative 3rd-line regimen [65]. In addition to the lower success rate compared with other candidates, the clinical impact is low if amoxicillin was included in a previous regimen, as the principle of salvage treatment is to use antibiotics that were not previously prescribed.

Potassium-competitive acid blockers (PCABs) have more potent and sustained acid-inhibitory effects than PPIs [66]. Vonoprazan showed excellent eradication success over 90% when used for 1st-line and 2nd-line regimens in a Japanese study [66]. Although clinical data regarding 3rd-line regimens containing PCAB are not available, promising results are expected [67].

## 6. Conclusions

With the growing antibiotic resistance worldwide, the role of a bacterial culture with antibiotic susceptibility testing and molecular susceptibility testing is crucial. However, susceptibility testing-guided treatment is not yet perfect. Therefore, providing patients with pretreatment medication instructions and education is important. Furthermore, the reason for the eradication failure should be determined and modified for a tailored therapy. Although the indications for *H. pylori* eradication have widened, patients at a high risk of gastric cancer can gain definitive benefits.
